# Protocol for a Randomized Controlled Trial to Evaluate a Permissive Blood Pressure Target Versus Usual Care in Critically Ill Children with Hypotension (PRESSURE)

**DOI:** 10.1097/PCC.0000000000003516

**Published:** 2024-04-17

**Authors:** Robert Darnell, Alanna Brown, Emma Laing, Julia Edwards, David A. Harrison, Joseph C. Manning, Mark J. Peters, Padmanabhan Ramnarayan, Samiran Ray, Zia Sadique, Barnaby R. Scholefield, Dermot Shortt, Lamprini Lampro, Carly Au, Kathy M. Rowan, Paul Mouncey, David P. Inwald

**Affiliations:** 1 Clinical Trials Unit, Intensive Care National Audit and Research Centre (ICNARC), London, United Kingdom.; 2 School of Healthcare, College of Life Sciences, University of Leicester, Leicester, United Kingdom.; 3 Infection, Immunity and Inflammation, University College London Great Ormond Street Institute of Child Health, London, United Kingdom.; 4 Department of Surgery and Cancer, Imperial College London, St Mary’s Campus, London, United Kingdom.; 5 Faculty of Public Health and Policy, London School of Hygiene and Tropical Medicine, London, United Kingdom.; 6 Department of Paediatric Critical Care Medicine, Hospital for Sick Children, University Avenue, Toronto, ON, Canada.; 7 Patient representative, c/o Clinical Trials Unit, Intensive Care National Audit and Research Centre (ICNARC), London, United Kingdom.; 8 Paediatric Intensive Care Unit, Addenbrooke’s Hospital, Cambridge University Hospitals NHS Foundation Trust, Cambridge, United Kingdom.

**Keywords:** inotrope, mean arterial pressure, pediatric intensive care, randomized clinical trial, vasopressor

## Abstract

**OBJECTIVES:**

Management of hypotension is a fundamental part of pediatric critical care, with cardiovascular support in the form of fluids or vasoactive drugs offered to every hypotensive child. However, optimal blood pressure (BP) targets are unknown. The PRotocolised Evaluation of PermiSSive BP Targets Versus Usual CaRE (PRESSURE) trial aims to evaluate the clinical and cost-effectiveness of a permissive mean arterial pressure (MAP) target of greater than a fifth centile for age compared with usual care.

**DESIGN:**

Pragmatic, open, multicenter, parallel-group randomized control trial (RCT) with integrated economic evaluation.

**SETTING:**

Eighteen PICUs across the United Kingdom.

**PATIENTS:**

Infants and children older than 37 weeks corrected gestational age to 16 years accepted to a participating PICU, on mechanical ventilation and receiving vasoactive drugs for hypotension.

**INTERVENTIONS:**

Adjustment of hemodynamic support to achieve a permissive MAP target greater than fifth centile for age during invasive mechanical ventilation.

**MEASUREMENTS AND MAIN RESULTS:**

Randomization is 1:1 to a permissive MAP target or usual care, stratified by site and age group. Due to the emergency nature of the treatment, approaching patients for written informed consent will be deferred until after randomization. The primary clinical outcome is a composite of death and days of ventilatory support at 30 days. Baseline demographics and clinical status will be recorded as well as daily measures of BP and organ support, and discharge outcomes. This RCT received Health Research Authority approval (reference 289545), and a favorable ethical opinion from the East of England—Cambridge South Research Ethics Committee on May 10, 2021 (reference number 21/EE/0084). The trial is registered and has an International Standard RCT Number (reference 20609635).

**CONCLUSIONS:**

Trial findings will be disseminated in U.K. national and international conferences and in peer-reviewed journals.

**RESEARCH IN CONTEXT**

- There is no high-quality evidence from randomized clinical trials comparing mean arterial pressure (MAP) targets in critically ill children.
- Emerging evidence from trials supports the use of permissive MAP targets in critically ill adults.
- The Protocolised Evaluation of Permissive Blood Pressure Targets Versus Usual Care trial will test the hypothesis that the benefits associated with a permissive MAP target will outweigh the risks in hypotensive critically ill children.

Hypotension is common in critically ill children and can compromise tissue perfusion and organ function. It is associated with increased risk of multiple organ failure and death after cardiac arrest and sepsis (1, 2). Hypotension is also associated with poor neurologic outcomes, particularly after cardiac arrest (1). In a review of 2005–2011 pediatric emergency practice in London, United Kingdom, the presence of septic shock at the time of critical care transport was associated with mortality of 20% (3).

Mean arterial pressure (MAP), reflecting perfusion pressure, is usually targeted in PICUs. Interventions to increase MAP may include IV fluids and vasoactive drugs. In the 2018 U.K. Paediatric Intensive Care Audit Network (PICANet, www.picanet.org.uk) dataset, around 30% of the 20,000 children admitted to U.K. PICUs received vasoactive drugs during their critical illness (4). Observational data from two London PICUs, obtained between 2009 and 2016, to look at usual practice (5), demonstrated that observed blood pressure (BP) was higher than 50th centile for age. However, though treating hypotension is beneficial, there are potential harms associated with the interventions. A systematic review of the literature from March 2017 showed an association between excessive IV fluids and prolonged PICU stay and increased mortality (6). Most vasoactive drugs cause vasoconstriction, which may reduce blood flow and impair end-organ function. Central venous catheters, sited to administer vasoactive drugs, are associated with thrombosis and infection, particularly in small children. The benefit-versus-harm balance when treating hypotension is complex, the threshold for harm is not clear, and a lower MAP than normal may be adequate. Aiming at a lower MAP than normal is referred to as “permissive hypotension.” Such a strategy echoes other permissive treatment strategies currently being evaluated in pediatric intensive care (7). The aim of the Protocolised Evaluation of Permissive BP Targets Versus Usual Care (PRESSURE) trial is to provide high-quality evidence to inform MAP targets in critically ill children.

In 2021, there was no high-quality evidence from randomized clinical trials (RCTs) comparing MAP targets in critically ill children. The 2020 Surviving Sepsis Campaign International Guidelines for the Management of Septic Shock and Sepsis-Associated Organ Dysfunction in Children suggested using a 5th–50th centile MAP target, while also noting the lack of RCT data in this area and highlighting the need for clinical trials (8). Advanced pediatric life support guidelines recommend targeting systolic BP at 5 mm Hg higher than the 50th centile without any trial-based evidence to support this practice. In the 1991–2018 pediatric literature, the study of BP targets in sepsis was, in general, part of retrospective examinations about bundles of care (9–14). In the adult patient literature about permissive hypotension, two separate RCTs were conducted during 2010–2011 (15) and 2013–2014 (16); when pooled, meta-analyses were carried out together in 2017 (17), suggesting that targeting higher MAP values of between 75 and 85 mm Hg may be associated with an increased risk of death in some older critically ill patients. The most recent RCT in adults recruited 2600 critically ill older patients with vasodilatory hypotension, in 2017–2019 (18), the “65 Trial,” showed no harm from a lower MAP target. There are no similar trial data in children. The PRESSURE trial will test the hypothesis that the benefits associated with a permissive MAP target in hypotensive critically ill children will outweigh the risks, improving outcomes and decreasing costs.

## MATERIALS AND METHODS

### Primary Objective

The primary objective is to estimate the clinical and cost-effectiveness of permissive MAP target (i.e., lower MAP target fifth centile for age) on a composite outcome of mortality and duration of invasive mechanical ventilation (IMV) at 30 days (i.e., rank-based analysis with death ranked as worse than 30 d of IMV), when compared with usual care.

### Design and Setting

PRESSURE is a pragmatic (19), multicenter, parallel-group RCT with integrated economic evaluation in infants and children accepted for admission to any of 18 National Health Service (NHS) PICUs across the United Kingdom and their regional retrieval services. It has previously been mentioned in the 2022 Paediatric Critical Care Society Study Group (PCCS-SG) summary of studies, published in *Pediatric Critical Care Medicine* (7).

### Screening and Randomization

Potentially eligible patients admitted (or accepted for admission) to participating PICU will be screened against the inclusion/exclusion criteria by the local clinical or transport team (**Table 1**). Patients will be randomized in a ratio 1:1 to either usual care or a permissive MAP target (fifth centile for age), stratified by site and age group (< 1, 1–3, ≥ 3 yr), to ensure balanced strata sizes. Randomization must occur within 6 hours of all the eligibility criteria being satisfied (Table 1). Randomization will be done by an automated randomization server accessible both by telephone and via web browser. The randomization sequence is computer-generated and will use variable block sizes to strengthen allocation concealment.

**TABLE 1.** Eligibility Criteria

Inclusion criteria
• Age > 37 wk corrected gestational age and < 16 yr
• Enrolled within 6 hr of first meeting all the following criteria;
◦ Accepted for or admitted to a participating PICU
◦ Face-to-face contact with PICU staff or transport team
◦ On invasive mechanical ventilation
◦ Receiving a continuous infusion of vasoactive drug for hypotension
◦ Vasoactive drug expected to continue for at least 6 hours or more^a^
Exclusion criteria
• Admitted postcardiac surgery
• Known cardiomyopathy^b^
• Neonates with suspected or proven duct-dependent circulation
• Acute brain injury^c^
• Currently being treated for pulmonary hypertension
• Admitted with malignant hypertension
• Death perceived as imminent
• Previously recruited to Protocolised Evaluation of Permissive Blood Pressure Targets Versus Usual Care trial

a “Vasoactive drug expected to continue for at least 6 hours or more” is at the discretion of the clinical team.

b “Known cardiomyopathy” refers to conditions known at time of randomization.

c “Acute brain injury” refers to traumatic brain injury or any acute or evolving neurologic condition requiring a neurointensive care strategy. In general, patients post cardiac arrest would not be eligible for randomization due to the high likelihood of acute brain injury. If the clinical team can rule out this contraindication (e.g., brief in-hospital cardiac arrest, when acute brain injury is not suspected) the patient would be eligible.

### Intervention and Concomitant Care

Following randomization, the allocated treatment will be commenced as soon as practically possible. In the intervention group, this means adjustment of vasoactive drugs and other therapies to achieve a MAP within the permissive MAP target band (lower MAP target fifth centile for age [20], see **Table 2**; and **Supplemental materials**, http://links.lww.com/PCC/C509 for details). In the usual care group, the MAP target is determined by the clinical team. The choice of treatments to achieve the MAP target and all other care is at the discretion of the clinical team.

**TABLE 2.** Protocolised Evaluation of Permissive Blood Pressure Targets Versus Usual Care Permissive Mean Arterial Pressure Target Bandings

Age Range (Completed Months/Yr)	Target Range (mm Hg)
37 wk–6 mo	40–43
> 6 mo–< 1 yr	40–45
≥ 1–3 yr	45–50
4–9 yr	50–55
≥ 10 yr	55–60

### Consent Procedures

Consent will be sought for the child (patient) from a parent/legal guardian with parental responsibility. Children become eligible for PRESSURE during a period of critical illness. Consequently, PRESSURE uses a “research without prior consent” model. Once a patient is screened and confirmed as eligible for the study, they will be randomized and the randomly assigned treatment will be commenced as soon as practically possible. This model, developed in line with the Consent Methods in Paediatric Emergency and Urgent Care Trials study guidance (21) has been found to be acceptable to parents/guardians, as well as to clinicians, in several PCCS-SG RCTs (22–26).

Once notified of the randomization of a patient into the study, a trained, delegated member of the site research team will approach parents/legal guardians of the patient as soon as possible to discuss the study, usually within 24–48 hours. A patient information sheet and consent form will be provided indicating: 1) the information has been read and understood, 2) consent is given for continuation in the trial, access to medical records, and linkage with routinely collected national data (e.g., national death registration data via NHS Digital or equivalent), 3) receipt of follow-up questionnaires, and 4) anonymized data to be shared in future.

A modification of the consent procedure will be used in two situations when either the patient: 1) is discharged from hospital before obtaining consent or 2) dies before consent is sought. In the former, the local research team will contact the parent/guardian, initially by telephone and then by post, for consent. If there is no response after 4 weeks, postal contact will be made again. If no consent form is received within 4 weeks of the second letter, the parent/guardian will be advised that the participant will be included in the trial unless they notify the research team otherwise. In the second situation, the local research team will establish the most appropriate clinical/research team member and time to notify the parents/guardians of involvement in the trial. If approach for consent before their departure from hospital is not deemed appropriate, then the same process as in the first situation, with contact by post, will be followed.

### Safety Monitoring

Adverse event (AE) reporting will follow the Health Research Authority guidelines on safety reporting in studies that do not involve investigational medicinal products (27). The following events have been prespecified as potential AEs that could be related to trial MAP target (or associated interventions): myocardial ischemia, arrhythmia, digital or limb ischemia, central line-associated bloodstream infection, thrombus related to central line insertion, skin necrosis related to administration of vasoactive via peripheral line, severe acute renal failure (as defined by the Kidney Disease Improving Global Outcomes criteria for stage 3), acute cerebral ischemia or infarction. These are reported if observed in participants from the time of randomization, for the duration of the treatment period, defined as the time the patient is in PICU during the acute hospital admission. This includes readmission to PICU from another inpatient care area (if the date of readmission is within 30 d from randomization).

Occurrences of specified, expected AEs will be recorded for all randomized patients. Considering that all infants and children eligible for PRESSURE are critically ill and at increased risk of experiencing AEs due to the complexity of their condition, occurrences of nonspecified AEs will only be reported if they are serious and are considered to be related to lower MAP values and/or higher doses of vasoactive agents required to maintain higher MAP values (i.e., “possibly,” “probably,” or “definitely”). Any event meeting these criteria will be considered a serious adverse event (SAE) and must be reported to the Intensive Care National Audit and Research Centre (ICNARC) Clinical Trials Unit (CTU). If the SAE is evaluated as a related and unexpected SAE, the ICNARC CTU will report to the research ethics committee (REC) within 15 calendar days.

## OUTCOME MEASURES

The primary clinical effectiveness outcome is a composite of mortality and duration of IMV, defined by the U.K. Paediatric Critical Care Minimum Dataset (28), from randomization to day 30. This will include any periods of reintubation. This outcome measure was chosen based on previous qualitative work which highlighted the child “looking and feeling more like themselves” and “time on machines” as the most important outcomes for parents (29). As intensive care interventions for cardiovascular support will be different between the intervention and control arms, the focus of the primary outcome measure is on respiratory support. Some other forms of organ support (e.g., renal replacement therapy) will be assessed as a secondary outcome measure. A full list of trial outcome measures can be found in **Table 3**. Due to the nature of the trial, it will not be possible to blind the outcome assessments.

**TABLE 3.** Trial Outcome Measures

Primary outcome—clinical effectiveness
• Composite of mortality and duration of invasive mechanical ventilation from randomization to day 30
Primary outcome—cost-effectiveness:
• Incremental net monetary benefit, evaluated at the U.K. National Instiute for Clinical Excellence recommended threshold of £20,000 per quality-adjusted life year at 12 mo
Secondary outcomes
• Mortality at PICU discharge, 30 d, 90 d, and 12 mo
• Duration of survival to 12 mo
• Time to first liberation from invasive ventilation
• Functional status change between PICU admission and PICU discharge, measured by the Pediatric Overall Performance Category and Pediatric Cerebral Performance Category scales (30)
• Receipt of renal replacement therapy at 30 d
• Length of PICU and hospital stay
• Health-related quality of life at 1 yr, measured by the child self- or parent-proxy reported Pediatric Quality of Life Inventory 4.0 (31) with age-appropriate versions covering the wide range included in the trial (1 mo–16 yr) and the Child Health Utility 9D Index (32)
• Incremental costs at 30 d

### Data Collection

Trial-specific data collection is limited to the minimum required to deliver trial objectives and will be collected at baseline (before randomization), daily, at discharge from PICU, and 30 days, 90 days, and 12 months following randomization (**Table 4**; and **Case Report Forms**, Supplementary Materials, http://links.lww.com/PCC/C509). The PRESSURE team will work closely with PICANet to make the best use of routinely collected PICU data, which includes baseline demographics, severity of illness scoring, and daily critical care interventions.

**TABLE 4.** Patient Data Collection Schedule

	Baseline (At Point of Randomization)	During Critical Care Unit Stay	End of Critical Care Unit Stay	At Hospital Discharge	At 1 yr
Patient details	✓				
Clinical/baseline data	✓				
Mean arterial pressure/vasopressors data	✓	✓	✓		
Cointerventions data		✓	✓		
Safety monitoring data		✓	✓		
Discharge data				✓	
Pediatric Quality of Life Inventory questionnaire (31)					✓
Child Health Utility 9D Index (32)					✓
Health services/resource use					✓

## STATISTICAL METHODS

### Sample Size

Mortality at 30 days and IMV days were estimated for the usual care arm using observational data from 4126 eligible sequential admissions to the FEVER study (investigating temperature thresholds for antipyretic intervention in critically ill children) (26), representing 39% of all unplanned admissions to U.K. NHS units reporting to PICANet in 2017 (4). Power estimates were based on 10,000 simulated trial datasets, using distributions of mortality and duration of IMV support (log-normal distribution with mean 7.8 and sd of 9.5 d). To achieve 90% power to detect a clinically meaningful reduction in mortality of 2% from 13% to 11% and the mean duration of IMV support of 18 hours from 7.8 to 7.1 days and allowing for 10% withdrawal/refusal of deferred consent requires a total sample size of 1900 patients. The same sample size will retain at least 80% power with either a mean reduction of 15 hours IMV support and a 2% reduction in mortality, or a mean reduction of 18 hours IMV support and a 1% reduction in mortality.

### Clinical Effectiveness Analysis

All analyses will be logged in a statistical analysis plan, a priori, before the investigators are unblinded to any study outcomes. All analyses will follow the intention-to-treat principle. Baseline patient characteristics will be compared between the two groups to observe balance and the success of randomization. These comparisons will not be subjected to statistical testing.

The analysis of the primary, composite, outcome will use rank-based methods, with death during the first 30 days following randomization ranked as the worst outcome and surviving patients ranked according to their duration of IMV support (30). The ranked outcomes will be compared between groups using a two-sample rank-sum (Wilcoxon-Mann-Whitney) test. The primary effect estimate will be the probabilistic index (the probability that the intervention is superior to the control for either mortality and/or duration of IMV support), which will be presented with a 95% CI. Duration of IMV support in surviving patients and mortality at 30 days will also be presented separately by arm with effect sizes and 95% CIs, in line with published guidelines for the use of composite primary endpoints (31).

Secondary analyses of mortality will be performed by Fisher exact test and adjusted logistic regression. Duration of survival to 12 months will be plotted as Kaplan-Meier survival curves, compared unadjusted with the log-rank test, and adjusted using Cox regression models. Time to first liberation from IMV will be analyzed by the log-rank test, with patients who die during IMV support treated as censored. Analyses of the length of PICU and hospital stay will be performed by rank-sum tests, stratified by survival status. Functional status will be assessed using the Pediatric Overall Performance Category and Pediatric Cerebral Performance Category scales (32). Healthcare-related quality of life (HRQoL) will be assessed using Pediatric Quality of Life Inventory (33) and Child Health Utility 9D Index (34) questionnaires at 12 months. Analyses of these data will be performed by *t*-tests and adjusted linear regression.

The primary endpoint and some secondary endpoints, will be reported in a limited number of prespecified clinically relevant subgroups, which will include patients classified by age. Results will be interpreted taking account of accepted criteria for credible subgroup effects (35, 36). A single interim analysis will be undertaken after recruitment and follow-up to 30 days of 50% of patients using a Peto-Haybittle stopping rule (*p* < 0.001) for termination due to either benefit or harm.

### Integrated Health Economic Evaluation

The cost-effectiveness analysis (CEA) will use patient-level resource use and outcome data collected as a part of the trial to assess the relative cost-effectiveness of permissive hypotension versus usual care strategies. The CEA will report the incremental cost-effectiveness results and summarize the joint uncertainties in incremental costs and health economic outcomes. The CEA will measure the costs in PICU, hospital, and broader health services costs. Patient-level resource use data from the index and subsequent readmissions to PICU and hospital admissions will be taken both from the case report form and linked to routine data from PICANet. Resource use data in broader healthcare services such as outpatient, primary, and community care will be collected by administering a patient follow-up Health Services Questionnaire, which will be developed as part of this study, at 12 months. Patient-level resource use data will be valued using the NHS Reference Costs and Personal Social Services Research Unit databases (37) to report total costs per patient for up to 12 months from randomization. HRQoL data at 12 months will be combined with survival data to report quality-adjusted life years (QALYs).

The CEA will follow the intention-to-treat principle and report the mean (95% CI) incremental costs, QALYs, and net monetary benefit at 12 months. The CEA will also perform a cost-consequence analysis and report incremental costs alongside primary outcome at 30 days. Missing data in costs and HRQoL will be handled using multiple imputation methods.

## GOVERNANCE AND OVERSIGHT

### Research Ethics

PRESSURE will be conducted in accordance with the approved trial protocol, the International Council for Harmonisation (ICH) of technical requirements for pharmaceuticals for human use, Good Clinical Practice (GCP) principles, the Data Protection Act (2018), the Helsinki Declaration of 1975, and ICNARC CTU research policies and procedures. This RCT, including its consent procedures, received Health Research Authority approval (IRAS reference 289545), and ethical approval from the East of England—Cambridge South REC on May 10, 2021 (REC reference number 21/EE/0084). The trial is registered on www.isrtcn.com and has an International Standard RCT number (reference 20609635).

### Confidentiality

Identifiable patient data, including name, contact details, date of birth, and NHS number, will be required by the ICNARC CTU to successfully follow-up participants. ICNARC CTU will act to preserve participant confidentiality and will not disclose or reproduce any information by which a participant could be identified. Data will be stored securely and accessed only by trained and authorized staff. ICNARC is registered under the Data Protection Act (registration number Z6289325), and all ICNARC CTU staff have undergone data protection and ICH-GCP training.

### Patient and Public Involvement

There was extensive patient and public involvement and engagement in the internal pilot phase, which informed the procedures for the main trial described here. Additionally, the parent of a child who received intensive care is among the investigator team and a member of the Trial Management Group (TMG), and another independent parent representative is a member of the Trial Steering Committee (TSC).

### Oversight

The TMG is responsible for the management of PRESSURE and meets regularly to monitor the conduct and progress of the trial. It is led by the Chief Investigator and includes the Investigators and the ICNARC CTU trial team. PRESSURE is managed by the ICNARC CTU in accordance with the Medical Research Council’s Good Research Practice: Principles and Guidelines (38), which is based on the ICH-GCP principles (39) and the U.K. Department of Health’s Policy Framework for Health and Social Care Research (40). The on-site monitoring plan follows a risk-based strategy. A majority independent TSC has been established to monitor trial progress. The committee is comprised of Patient and Public Involvement representatives, experienced clinicians and researchers, the Chief Investigator, and the Head of Research at ICNARC. An independent Data Monitoring and Ethics Committee has been established to monitor patient recruitment and retention, adherence, and safety. Cambridge University Hospitals NHS Foundation Trust is the trial sponsor (reference A095842). As the sponsor is an NHS organization, NHS indemnity will apply for legal liability arising from the design, management, and conduct of the research.

### Trial Status

This article presents the study protocol (v4.0), dated August 25, 2022, accessible at www.icnarc.org. Due to the COVID-19 pandemic, recruitment (which was originally scheduled to commence in February 2021) was postponed until August 2021. The trial was also paused between September 2021 and November 2021 (before any participants were recruited) to review the protocol. The first participant was recruited in November 2021. The trial was assessed by the funder after the internal pilot phase (first 6 mo of the recruitment period) and progressed to a full trial: at the time of submission patient recruitment was ongoing. The study will be disseminated through publication in peer-reviewed medical journals and at national and international conferences.

## ACKNOWLEDGMENTS

The authors thank the research and clinical teams at the participating sites: Addenbrooke’s Hospital (Cambridge), Alder Hey Children’s Hospital (Liverpool), Birmingham Women and Children’s Hospital, Bristol Royal Hospital for Children, Great North Children’s Hospital (Newcastle), Great Ormond Street Hospital (London), John Radcliffe Hospital (Oxford), King’s College Hospital (London), Leeds Children’s Hospital, Leicester Royal Infirmary, Noah’s Ark Children’s Hospital (Cardiff), Queen’s Medical Centre (Nottingham), Royal Hospital for Children (Glasgow), The Royal London Hospital, Royal Manchester Children’s Hospital, Sheffield Children's Hospital, Southampton Children’s Hospital, St George’s Hospital (London), and St Mary’s Hospital (London). The authors also thank the clinical and administrative staff of the following transport teams for their support for this trial: Children’s Acute Transport Service, Children’s Medical Emergency Transport, Embrace Transport, KIDS Combined Neonatal and Paediatric Critical Care Advice and Transport Service, North East & Cumbria Transport and Retrieval, North West and North Wales Paediatric Transport Service, Paediatric and Neonatal Decision Support and Retrieval Service, Southampton Oxford Retrieval Team and Wales and West Acute Transport for Children. The authors thank Mr. Dermot Shortt, Patient and Public Involvement representative, for his valued contribution to this work.

## Supplementary Material
