# Supplementary material for: Protocol for a Randomized Controlled Trial to Evaluate a Permissive Blood Pressure Target Versus Usual Care in Critically Ill Children with Hypotension (PRESSURE)
Source: Pediatr Crit Care Med. 2024 Apr 17;25(7):629–37. doi: 10.1097/PCC.0000000000003516 (PMC11216373; doi:10.1097/PCC.0000000000003516)
Supplement: Supplementary file 1 [file pcc-25-629-s001.pdf]

Supplementary material

Table of contents

The development of the permissive mean arterial pressure target ranges in PRESSURE – p.3 – p.6

Case Report Form – p.7 – p.23

The development of the permissive mean arterial pressure target ranges in PRESSURE

Introduction

It is usual practice amongst pediatric intensivists to define a mean arterial BP (MAP) target when patients are critically unwell – since this is the determinant of organ perfusion. These targets drive therapeutic interventions by bedside staff. The PRESSURE trial aims to compare the safety and efficacy of a lower MAP target in children on vasoactive drugs on the PICU; specifically, a 5th centile MAP target with standard care. The study team needed therefore both to define 5th centile MAP for every child entered into the study and to present this target in a pragmatic format for clinical intensive care staff.

MAP centile ranges were reported by Haque and Zaritsky in *Pediatric Critical Care Medicine* in 2008; these are still widely referenced and used today (1). These centile ranges were derived using data from the US National Institutes for Health (NIH) “Fourth Report on the Diagnosis, Evaluation, and Treatment of High Blood Pressure in Children and Adolescents”, (“Task Force data”) from healthy children between 1 and ≤18 yrs old (2).

In developing the PRESSURE 5th centile permissive target, the study team considered it important to take account of the retrospective studies by Topjian *et al.* These demonstrated that children post cardiac arrest who had systolic BP (SBP) <5th centile in the first 6 hours had worse outcome (3,4). The SBP centiles used by Topjian *et al* were also derived from an analysis of the NIH Task Force data, but excluding overweight children (5), resulting in centiles lower than previously published Task Force centiles.

There are few observational population studies which have presented 5th centile MAP in healthy children. MAP centiles derived from studies undertaken in hospital, using data acquired either non-invasively or invasively, in intensive care or in a ward environment, are different to those reported by Haque and Zaritsky (6,7,8). It is unclear why this is the case, though it may be due to differences in measurement methodology; and in two of the hospital studies in which higher pressures were observed, this may have been due to disease, patient distress or therapeutic interventions (7,8).

The PRESSURE study team therefore chose to base the PRESSURE target range around the Haque and Zaritsky 5th centile MAP for a number of reasons: a) it is based on Task Force data, derived from measurements in healthy children outside hospital, and therefore unlikely to be confounded by disease or hospital treatment; b) it is the data upon which the

Paediatric Sepsis Consensus Conference based their definition of systolic hypotension (9) and also referenced in numerous guidelines; and c) Topjian *et al* used centiles derived from Task Force data in their studies looking at outcome post cardiac arrest.

However, the European Resuscitation Council (ERC) released a paper just prior to the opening of the PRESSURE study, which included a table of 5th centile MAPs (10). These were based on one of the in-hospital studies cited above, a single centre study reporting MAP centiles derived from invasive arterial blood pressure (IABP) measurements taken from children in PICU (6). The view of the clinical co-Investigators in the PRESSURE Trial Management Group was that IABP data from children in PICU with arterial access was likely to be confounded by treatment effect. Despite this, the group acknowledged the ERC guidance and feedback from clinical teams at some study sites around the lower MAP target in younger children. The group concluded that the PRESSURE target ranges should not be below the ERC 5th centile.

Thus, for infants under 1 year of age, where Task Force data are not available, and observational population based studies are limited, the lower limit of the PRESSURE permissive target range was based on the ERC 5th centile. For older children, where Task Force data were available, the PRESSURE permissive target range was based around the Haque and Zaritsky 5th centile MAP (based on an average of male and female 5th centile MAP, for children of 50th centile height) but never lower than the ERC 5th centile. There is relatively little difference in MAP between genders and height is often not measured in a PICU setting, so the PRESSURE permissive MAP target is presented in age bands, as ranges with upper and lower limits, easily understood by bedside staff (Supplementary Figure and Table 2, main manuscript).

Figure

PRESSURE permissive MAP target bandings (green shaded area), presented with 5th centile MAP from Haque and Zaritsky data (1) (averaged male/female for 50th centile height) and 5th centile MAP from the ERC table (10).

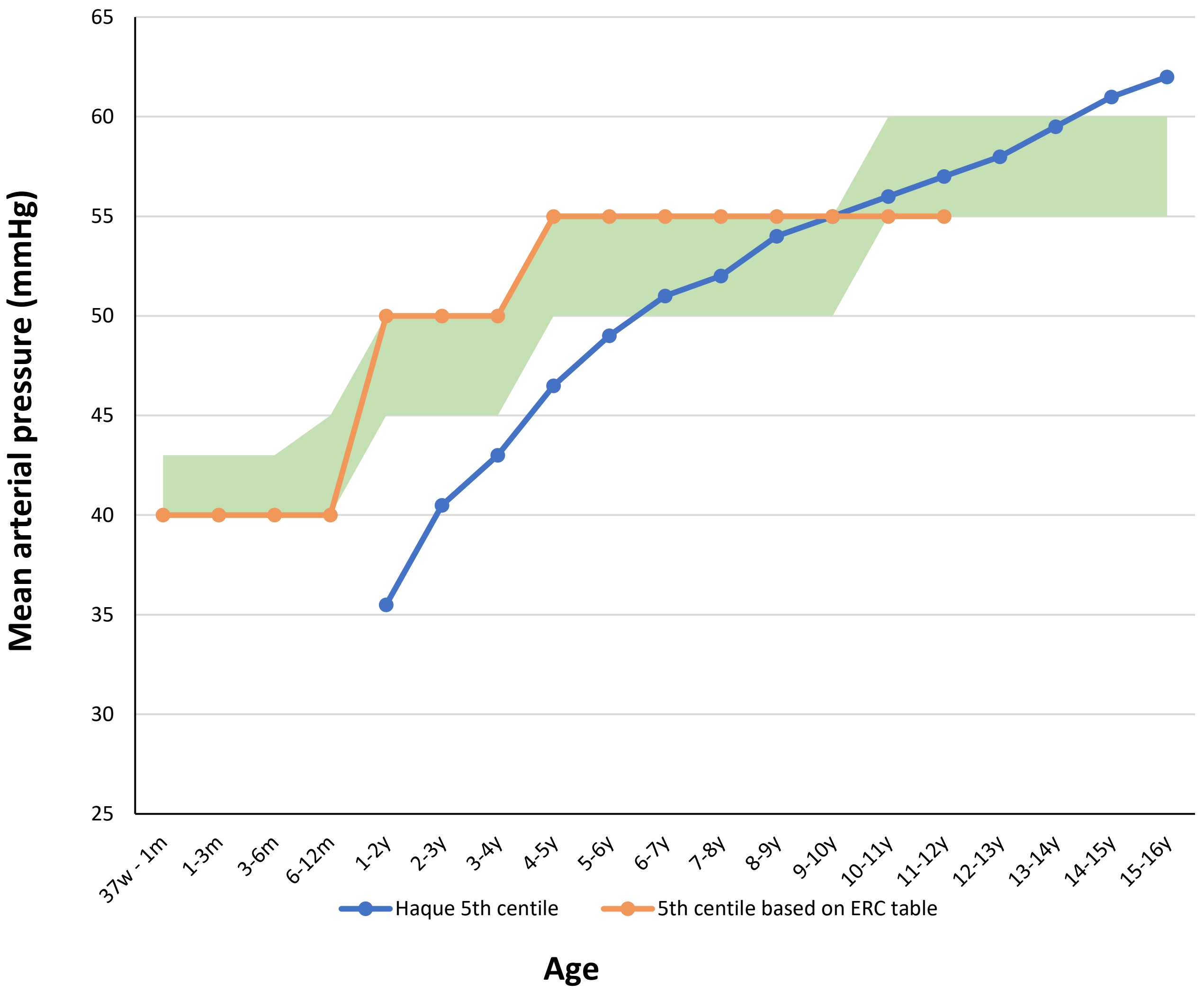

References

1. Haque IU, Zaritsky AL. Analysis of the evidence for the lower limit of systolic and mean arterial pressure in children. Pediatr Crit Care Med. 2007;8:138 –144
2. National High Blood Pressure Education Program Working Group on High Blood Pressure in Children and Adolescents. The Fourth Report on the Diagnosis, Evaluation, and Treatment of High Blood Pressure in Children and Adolescents. Pediatrics 2004;114:555-576
3. Topjian AA, French B, Sutton RM, et al. Early postresuscitation hypotension is associated with increased mortality following pediatric cardiac arrest. Crit Care Med. 2014;42:1518–1523
4. Topjian AA, Telford R, Holubkov R, et al. Association of early postresuscitation pypotension with survival to discharge after targeted temperature management for pediatric out-of-hospital cardiac arrest: secondary analysis of a randomized clinical trial. JAMA Pediatr. 2018;172:143-153.
5. Rosner B, Cook N, Portman R et al. Determination of blood pressure percentiles in normal-weight children: some methodological issues. Am J Epidemiol 2008;167:653–666
6. Eytan D, Goodwin AJ, Greer R, Guerguerian A-M and Laussen PC. Heart Rate and Blood Pressure Centile Curves and Distributions by Age of Hospitalized Critically Ill Children. Front. Pediatr. 2017;5:52
7. Abdelrazeq S, Ray S, Rogers L et al. Age-associated blood pressure distributions in paediatric intensive care units differ from healthy children. Intensive Care Med 2018;44:384–386
8. Roberts JS, Yanay O, Barry D. Age-Based Percentiles of Measured Mean Arterial Pressure in Pediatric Patients in a Hospital Setting. Pediatr Crit Care Med. 2020;21:e759-e768
9. Goldstein B, Giroir B, Randolph A; International Consensus Conference on Pediatric Sepsis. International pediatric sepsis consensus conference: definitions for sepsis and organ dysfunction in pediatrics. Pediatr Crit Care Med. 2005;6:2-8
10. Van de Voorde P, Turner NM, Djakow J et al. European Resuscitation Council Guidelines 2021: Paediatric Life Support. Resuscitation. 2021;161:327-387

Data Collection Worksheets

Please ensure CRFs stay with patient

Treatment allocation

Permissive Blood Pressure Target (P)

Usual Care (C)

Permissive Blood Pressure Targets

Select	Age range (completed months/years)	Target Range
☐	Less than 6 months	40 - 43
☐	6 months - less than 1 year	40 - 45
☐	1 - 3 years	45 - 50
☐	4 - 9 years	50 - 55
☐	≥10 years	55 - 60

Trial ID

Date / Time of Randomisation

Date: D D / M M / 2 0 2 Y   Time: H H : M M (24-hour clock)

Baseline: Demographics/Observations

Trial ID

PRESSURE

Demographics

Sex: Male ○ Female ○

Hospital number:

Weight: ___ . _ kg (Estimated (E) / Measured (M))

PICANet event ID:

Physiology/Interventions

Last observations prior to randomisation

Values must be recorded within the hour prior to randomisation.

			Not recorded
Arterial PaO_2:	___ . _	kPa / mmHg *(Delete as appropriate)*	○
Base excess:	+/- __ . _	mmol l^{-1} (Arterial (A) / Capillary (C) / Venous (V))	○
Lactate:	__ . _	mmol l^{-1} (Arterial (A) / Capillary (C) / Venous (V))	○
FiO_2: (decimal)	_ . __		○

Pupil reaction:	Not recorded
Both equal and reactive ○	○
Both fixed and dilated ○	
Other reaction ○	
Unknown ○	

Systolic BP: ___ mmHg ○ (Not recorded)

Vasoactive drugs at randomisation (if received)

When did the patient commence continuous infusion of vasoactive drug(s) for hypotension?

Date/Time: D D / M M / 2 0 2 Y   H H : M M (24-hour clock)

Drug	Entry	Units
Noradrenaline / Norepinephrine:	_ . __	mcg/kg/min
Adrenaline / Epinephrine:	_ . __	mcg/kg/min
Dopamine:	__	mcg/kg/min
Dobutamine:	__	mcg/kg/min
Metaraminol:	__ . __	mcg/kg (bolus) **OR** mcg/h (infusion) ***(Delete as appropriate)***
Vasopressin:	_ . ____	units/kg/min
Terlipressin:	__ . _	mcg/kg (bolus) **OR** mcg/kg/h (infusion) ***(Delete as appropriate)***
Milrinone:	_ . __	mcg/kg/min
Phenylephrine:	__ . __	mcg/kg (bolus) **OR** mcg/kg/min (infusion) ***(Delete as appropriate)***

Completed by:
(print name)

Signature:

Date completed: D D / M M / 2 0 2 Y

Baseline: Comorbidities

Trial ID | | | | | |

PRESSURE

Documented pre-existing conditions that existed in the 12 months preceding this admission to the PICU

**Cardiac/Vascular** (e.g. Atrioventricular septal defect, Tetralogy of Fallot)	Yes ◯ No ◯	**Gastro/Surgical** (e.g. Tracheo-oesophageal fistula, Gastoschisis)	Yes ◯ No ◯
**Congenital/Genetic/Syndrome** (e.g. Trisomy 21, Dravet syndrome)	Yes ◯ No ◯	**Haematology/Oncology** (e.g. Acute leukaemia, Medulloblastoma)	Yes ◯ No ◯
**Neurological/Neuromuscular** (e.g. Cerebral Palsy, Spinal muscular atrophy)	Yes ◯ No ◯	**Metabolic/Endrocrine** (e.g. Diabetes Type I, Hypothyroidism)	Yes ◯ No ◯
**Immunodeficiency** (Characterised by a chronic state of a reduced ability to resist infection for at least three months as a result of a primary diagnosis, therapy or combination of both)	Yes ◯ No ◯	**Airway/Respiratory** (e.g. Chronic lung disease, Asthma)	Yes ◯ No ◯
**End Stage Kidney Disease on Renal Replacement Therapy**	Yes ◯ No ◯	**Other** If other, specify;	Yes ◯ No ◯

Current Admission

Infection as cause of current admission — Suspected ◯ Proven ◯ N/A ◯

Cardiac arrest prior to randomisation* — Yes ◯ No ◯

*requires either documented absent pulse or the requirement for external cardiac compression

Completed by:
(print name)

Signature:

Date completed: D D / M M / 2 0 2 Y

Functional status prior to PICU admission

Date of Assessment D D / M M / 2 0 2 Y

Trial ID

Not applicable, patient died NA

Paediatric Cerebral Performance Category (PCPC)

**Score** (Select One)	**Category**	**Description**
1	Normal	Age-appropriate level of functioning<br>Pre-school child: developmentally appropriate<br>School-aged child: can attend regular school classroom
2	Mild disability	Conscious, alert and able to interact at age-appropriate level<br>Pre-school child: may have minor developmental delays<br>School-aged child: can attend regular classroom, but grade is not appropriate for age, or child is likely to fail appropriate grade because of cognitive difficulties
3	Moderate disability	Conscious, below age-appropriate functioning<br>Neurologic disease that is not controlled and severely limits activities<br>Pre-school child: delayed for most of their activities of daily living<br>School-aged child: Sufficient cerebral function for age-appropriate independent activities of daily life, must attend special education classroom because of cognitive difficulties and/or learning deficits
4	Severe disability	Conscious<br>Pre-school child: delayed for most of their activities of daily living and excessively dependent on others for the provision of activities of daily living<br>School-aged child: may be so impaired as to be unable to attend school, dependent on others for daily support because of impaired brain function
5	Coma or vegetative state	Any degree of coma<br>Unaware, even if awake in appearance, without interaction with environment<br>Cerebral unresponsiveness and no evidence of cortical function (eg. not aroused by verbal stimuli)<br>Possibility of some reflective response, spontaneous eye-opening and sleep-wake cycles

Paediatric Overall Performance Category (POPC)

**Score** (Select One)	**Category**	**Description**
1	Good overall performance	PCPC is classified as Normal<br>Healthy, alert and capable of normal age-appropriate activities of daily life<br>Medical and physical problems do not interfere with normal activity
2	Mild overall disability	PCPC classified as Mild Disability<br>Minor chronic physical or medical problems present minor limitations but are compatible with normal life (eg. asthma)<br>Pre-school child: has a physical disability consistent with future independent functioning (eg. a single amputation) and is able to perform the majority of age-appropriate activities of daily living
3	Moderate overall disability	PCPC classified as Moderate Disability<br>Possibility of moderate disability from non-cerebral systems dysfunction alone or with cerebral system dysfunction<br>Pre-school child: delayed for most of their activities of daily living<br>School-aged child: conscious and performs independent activities of daily life but is physically disabled (eg. cannot participate in competitive physical activities)
4	Severe overall disability	PCPC classified as Severe Disability<br>Possibility of severe disability from non-cerebral systems dysfunction alone or with cerebral system dysfunction<br>Pre-school child: delayed for most of their activities of daily living <u>and</u> excessively dependent on others for the provision of activities of daily living<br>School-aged child: dependent on others for most activities of daily living
5	Coma or vegetative state	PCPC classified as coma/vegetative

Signature | **Completed by** (print name) | **Date completed:** D D / M M / 2 0 2 Y

Observations: Day 1 (day of randomisation)

Trial ID: | | | | | |

Data collection should start on the first whole hour after randomisation (e.g. patient randomised at 08:40, data collection should begin at 09:00).

Date: D D / M M / 2 0 2 Y

Part 1

Hourly values (00:00 – 12:00)	00:00	01:00	02:00	03:00	04:00	05:00	06:00	07:00	08:00	09:00	10:00	11:00	12:00
MAP (mmHg)													
**On Vasoactives?**	Y N	Y N	Y N	Y N	Y N	Y N	Y N	Y N	Y N	Y N	Y N	Y N	Y N
Noradrenaline/ Norepinephrine mcg/kg/min													
Adrenaline/ Epinephrine mcg/kg/min													
Dopamine mcg/kg/min													
Dobutamine mcg/kg/min													
Milrinone mcg/kg/min													
Vasopressin units/kg/min													
Terlipressin mcg/kg (bolus) **OR** mcg/kg/h (infusion) *(delete as appropriate)*													
Phenylephrine mcg/kg (bolus) **OR** mcg/kg/h (infusion) *(delete as appropriate)*													

Guidance

Usual Care Patients

- Data collection should continue until the patient is off vasoactives for 24 consecutive hours (i.e. 'No' for 24 consecutive hours) or until discharge from PICU.
- If vasoactives are restarted after 24 consecutive hours during the hospital admission, this is defined as a separate episode and should be recorded on the 'Outcomes' page.

Permissive Blood Pressure Target Patients

- Data collection should continue until the patient is off vasoactives for 24 consecutive hours (i.e. 'No' for 24 consecutive hours) or until discharge from PICU.
- The Permissive Blood Pressure Target will apply at any point the patient requires vasoactive drugs whilst on PICU during the acute hospital admission.
- If vasoactives are restarted after 24 consecutive hours during the hospital admission, this is defined as a separate episode and should be recorded on the 'Outcomes' page.

	Age range (completed months/years)	Target range
☐	Less than 6 months	40 - 43
☐	6 months - less than 1 year	40 - 45
☐	1 - 3 years	45 - 50
☐	4 - 9 years	50 - 55
☐	≥10 years	55 - 60

Signature: ____ **Completed by:** (print name) ____ **Date completed:** D D / M M / 2 0 2 Y

Observations: Day 1

Data collection should start on the first whole hour after randomisation (e.g. patient randomised at 08:40, data collection should begin at 09:00)

Trial ID | | | | | | |

PRESSURE

Part 2

Additional pages

Date: D D / M M / 2 0 2 Y

Hourly values (13:00 – 23:00)

	13:00	14:00	15:00	16:00	17:00	18:00	19:00	20:00	21:00	22:00	23:00
MAP (mmHg)											
**On Vasoactives?**	Y N	Y N	Y N	Y N	Y N	Y N	Y N	Y N	Y N	Y N	Y N
Noradrenaline/ Norepinephrine mcg/kg/min											
Adrenaline/ Epinephrine mcg/kg/min											
Dopamine mcg/kg/min											
Dobutamine mcg/kg/min											
Milrinone mcg/kg/min											
Vasopressin units/kg/min											
Terlipressin mcg/kg (bolus) **OR** mcg/kg/h (infusion) *(delete as appropriate)*											
Phenylephrine mcg/kg (bolus) **OR** mcg/kg/h (infusion) *(delete as appropriate)*											

Guidance

Usual Care Patients

- Data collection should continue until the patient is off vasoactives for 24 consecutive hours (i.e. 'No' for 24 consecutive hours) or until discharge from PICU.
- If vasoactives are restarted after 24 consecutive hours during the hospital admission, this is defined as a separate episode and should be recorded on the 'Outcomes' page.

Permissive Blood Pressure Target Patients

- Data collection should continue until the patient is off vasoactives for 24 consecutive hours (i.e. 'No' for 24 consecutive hours) or until discharge from PICU.
- The Permissive Blood Pressure Target will apply at any point the patient requires vasoactive drugs whilst on PICU during the acute hospital admission.
- If vasoactives are restarted after 24 consecutive hours during the hospital admission, this is defined as a separate episode and should be recorded on the 'Outcomes' page.

	Age range (completed months/years)	Target range
☐	Less than 6 months	40 - 43
☐	6 months - less than 1 year	40 - 45
☐	1 - 3 years	45 - 50
☐	4 - 9 years	50 - 55
☐	≥10 years	55 - 60

Please use additional pages to document further observations

Signature: ______ **Completed by:** (print name) ______ **Date completed:** D D / M M / 2 0 2 Y

Observations: Day _

Trial ID | | | | | | |

PRESSURE

Data collection should start on the first whole hour after randomisation (e.g. patient randomised at 08:40, data collection should begin at 09:00)

Date: D D / M M / 2 0 2 Y

Part 1

Additional pages

Hourly values (00:00 – 12:00)	00:00	01:00	02:00	03:00	04:00	05:00	06:00	07:00	08:00	09:00	10:00	11:00	12:00
MAP (mmHg)													
**On Vasoactives?**	Y ↓ N →	Y ↓ N →	Y ↓ N →	Y ↓ N →	Y ↓ N →	Y ↓ N →	Y ↓ N →	Y ↓ N →	Y ↓ N →	Y ↓ N →	Y ↓ N →	Y ↓ N →	Y ↓ N →
Noradrenaline/ Norepinephrine mcg/kg/min													
Adrenaline/ Epinephrine mcg/kg/min													
Dopamine mcg/kg/min													
Dobutamine mcg/kg/min													
Milrinone mcg/kg/min													
Vasopressin units/kg/min													
Terlipressin mcg/kg (bolus) **OR** mcg/kg/h (infusion) *(delete as appropriate)*													
Phenylephrine mcg/kg (bolus) **OR** mcg/kg/h (infusion) *(delete as appropriate)*													

Guidance

Usual Care Patients

- Data collection should continue until the patient is off vasoactives for 24 consecutive hours (i.e. 'No' for 24 consecutive hours) or until discharge from PICU.
- If vasoactives are restarted after 24 consecutive hours during the hospital admission, this is defined as a separate episode and should be recorded on the 'Outcomes' page.

Permissive Blood Pressure Target Patients

- Data collection should continue until the patient is off vasoactives for 24 consecutive hours (i.e. 'No' for 24 consecutive hours) or until discharge from PICU.
- The Permissive Blood Pressure Target will apply at any point the patient requires vasoactive drugs whilst on PICU during the acute hospital admission.
- If vasoactives are restarted after 24 consecutive hours during the hospital admission, this is defined as a separate episode and should be recorded on the 'Outcomes' page.

	**Age range** (completed months/years)	**Target range**
☐	Less than 6 months	40 - 43
☐	6 months - less than 1 year	40 - 45
☐	1 - 3 years	45 - 50
☐	4 - 9 years	50 - 55
☐	≥10 years	55 - 60

Signature: ______ **Completed by:** (print name) ______ **Date completed:** D D / M M / 2 0 2 Y

Observations: Day _ (day of randomisation)

Trial ID | | | | | | |

PRESSURE

Data collection should start on the first whole hour after randomisation (e.g. patient randomised at 08:40, data collection should begin at 09:00)

Date: D D / M M / 2 0 2 Y

Part 2
Additional Pages

Hourly values (13:00 – 23:00)	13:00	14:00	15:00	16:00	17:00	18:00	19:00	20:00	21:00	22:00	23:00
MAP (mmHg)											
**On Vasoactives?**	Y N	Y N	Y N	Y N	Y N	Y N	Y N	Y N	Y N	Y N	Y N
Noradrenaline/ Norepinephrine mcg/kg/min											
Adrenaline/ Epinephrine mcg/kg/min											
Dopamine mcg/kg/min											
Dobutamine mcg/kg/min											
Milrinone mcg/kg/min											
Vasopressin units/kg/min											
Terlipressin mcg/kg (bolus) **OR** mcg/kg/h (infusion) *(delete as appropriate)*											
Phenylephrine mcg/kg (bolus) **OR** mcg/kg/h (infusion) *(delete as appropriate)*											

Guidance

Usual Care Patients

- Data collection should continue until the patient is off vasoactives for 24 consecutive hours (i.e. 'No' for 24 consecutive hours) or until discharge from PICU.
- If vasoactives are restarted after 24 consecutive hours during the hospital admission, this is defined as a separate episode and should be recorded on the 'Outcomes' page.

Permissive Blood Pressure Target Patients

- Data collection should continue until the patient is off vasoactives for 24 consecutive hours (i.e. 'No' for 24 consecutive hours) or until discharge from PICU.
- The Permissive Blood Pressure Target will apply at any point the patient requires vasoactive drugs whilst on PICU during the acute hospital admission.
- If vasoactives are restarted after 24 consecutive hours during the hospital admission, this is defined as a separate episode and should be recorded on the 'Outcomes' page.

	**Age range** (completed months/years)	**Target range**
☐	Less than 6 months	40 - 43
☐	6 months - less than 1 year	40 - 45
☐	1 - 3 years	45 - 50
☐	4 - 9 years	50 - 55
☐	≥10 years	55 - 60

Signature: ____________ **Completed by:** (print name) ____________ **Date completed:** D D / M M / 2 0 2 Y

Daily Values

Trial ID | | | | | |

Record daily values for each day from randomisation up to 30 days whilst the patient is in PICU

Day 1 = calculate daily fluid balance from time of randomisation until standard unit charting time (e.g. if the participant was randomised at 05:00 and your unit's standard charting time is 08:00 – 08:00, Day 1 should include data from 05:00 – 08:00)".
Day 2 onwards = log data at standard unit charting time for 24 hours fluid balance

Fluid bolus = any intravenous fluid given as rapid volume expansion

	Day 1	Day 2 NA	Day 3 NA	Day 4 NA	Day 5 NA	Day 6 NA	Day 7 NA	Day 8 NA	Day 9 NA	Day 10 NA
	DD/MM/2Y	DD/MM/2Y	DD/MM/2Y	DD/MM/2Y	DD/MM/2Y	DD/MM/2Y	DD/MM/2Y	DD/MM/2Y	DD/MM/2Y	DD/MM/2Y
Fluid bolus received	Yes (Y) No (N)	Yes (Y) No (N)	Yes (Y) No (N)	Yes (Y) No (N)	Yes (Y) No (N)	Yes (Y) No (N)	Yes (Y) No (N)	Yes (Y) No (N)	Yes (Y) No (N)	Yes (Y) No (N)
Fluid bolus volume (ml)										
Daily fluid balance (ml)	+/-	+/-	+/-	+/-	+/-	+/-	+/-	+/-	+/-	+/-
Daily urine output (ml)										

	Day 11 NA	Day 12 NA	Day 13 NA	Day 14 NA	Day 15 NA	Day 16 NA	Day 17 NA	Day 18 NA	Day 19 NA	Day 20 NA
	DD/MM/2Y	DD/MM/2Y	DD/MM/2Y	DD/MM/2Y	DD/MM/2Y	DD/MM/2Y	DD/MM/2Y	DD/MM/2Y	DD/MM/2Y	DD/MM/2Y
Fluid bolus received	Yes (Y) No (N)	Yes (Y) No (N)	Yes (Y) No (N)	Yes (Y) No (N)	Yes (Y) No (N)	Yes (Y) No (N)	Yes (Y) No (N)	Yes (Y) No (N)	Yes (Y) No (N)	Yes (Y) No (N)
Fluid bolus volume (ml)										
Daily fluid balance (ml)	+/-	+/-	+/-	+/-	+/-	+/-	+/-	+/-	+/-	+/-
Daily urine output (ml)										

	Day 21 NA	Day 22 NA	Day 23 NA	Day 24 NA	Day 25 NA	Day 26 NA	Day 27 NA	Day 28 NA	Day 29 NA	Day 30 NA
	DD/MM/2Y	DD/MM/2Y	DD/MM/2Y	DD/MM/2Y	DD/MM/2Y	DD/MM/2Y	DD/MM/2Y	DD/MM/2Y	DD/MM/2Y	DD/MM/2Y
Fluid bolus received	Yes (Y) No (N)	Yes (Y) No (N)	Yes (Y) No (N)	Yes (Y) No (N)	Yes (Y) No (N)	Yes (Y) No (N)	Yes (Y) No (N)	Yes (Y) No (N)	Yes (Y) No (N)	Yes (Y) No (N)
Fluid bolus volume (ml)										
Daily fluid balance (ml)	+/-	+/-	+/-	+/-	+/-	+/-	+/-	+/-	+/-	+/-
Daily urine output (ml)										

Signature: ________ **Completed by:** (print name) ________ **Date completed:** DD/MM/202Y

Organ Support Daily Observations

Please tick all types of organ support a patient receives on a given day for any length of time whilst in PICU, or 'no support' if none.

Trial ID | | | | | | |

Day 1 = from time of randomisation to midnight
Day 2 – 30 = calendar days

Organ Support		Day 1<br>D D / M M / 2 Y<br>☐ Not in PICU	Day 2<br>D D / M M / 2 Y<br>☐ Not in PICU	Day 3<br>D D / M M / 2 Y<br>☐ Not in PICU	Day 4<br>D D / M M / 2 Y<br>☐ Not in PICU	Day 5<br>D D / M M / 2 Y<br>☐ Not in PICU	Day 6<br>D D / M M / 2 Y<br>☐ Not in PICU	Day 7<br>D D / M M / 2 Y<br>☐ Not in PICU
No Support								
Respiratory	Invasive respiratory support							
	Any NIV (including HFNC)**							
Cardiovascular Interventions								
Corticosteroids given for cardiovascular instability								
Mechanical cardiovascular support								
Renal replacement therapy								
Plasma filtration or exchange								

		Day 8<br>D D / M M / 2 Y<br>☐ Not in PICU	Day 9<br>D D / M M / 2 Y<br>☐ Not in PICU	Day 10<br>D D / M M / 2 Y<br>☐ Not in PICU	Day 11<br>D D / M M / 2 Y<br>☐ Not in PICU	Day 12<br>D D / M M / 2 Y<br>☐ Not in PICU	Day 13<br>D D / M M / 2 Y<br>☐ Not in PICU	Day 14<br>D D / M M / 2 Y<br>☐ Not in PICU	Day 15<br>D D / M M / 2 Y<br>☐ Not in PICU
No Support									
Respiratory	Invasive respiratory support								
	Any NIV (including HFNC)**								
Cardiovascular Interventions									
Corticosteroids given for cardiovascular instability									
Mechanical cardiovascular support									
Renal replacement therapy									
Plasma filtration or exchange									

** NIV= Non Invasive Ventilation
HFNC = High Flow Nasal Cannula

DEFINITIONS: ORGAN SUPPORT

Invasive Respiratory Support
- Invasive mechanical ventilation i.e. must be intubated and ventilated
- Includes mechanical ventilation via an endotracheal tube or via tracheostomy
- Includes High Frequency Oscillation Ventilation (HFOV)

Any NIV (Non-invasive mechanical Ventilation)
- CPAP / BiPAP via mask (facial or nasal mask)
- High Flow Nasal Oxygen via nasal cannula (HFNC)

Do not include (in either group)
- Nasopharyngeal airway without ventilation
- Supplementary oxygen therapy alone

Cardiovascular
- Continuous inotrope/vasodilator/ prostaglandin infusion
- CPR
- Anti-arrhythmic therapy

Corticosteroids given for cardiovascular instability

Mechanical Cardiovascular Support
- ECMO
- Vascular assist device
- Aortic balloon pump

Renal Replacement Therapy
- Peritoneal dialysis
- Haemofiltration
- Haemodialysis

Plasma filtration or exchange

**Signature:**		**Completed by:** (print name)		**Date completed:**	D D / M M / 2 0 2 Y

Organ Support Daily Observations

Please tick all types of organ support a patient receives on a given day for any length of time whilst in PICU, or 'no support' if none.

Trial ID | | | | | | |

Day 1 = from time of randomisation to midnight
Day 2 – 30 = calendar days

**Organ Support**		Day 16<br>D D / M M / 2 Y<br>☐ Not in PICU	Day 17<br>D D / M M / 2 Y<br>☐ Not in PICU	Day 18<br>D D / M M / 2 Y<br>☐ Not in PICU	Day 19<br>D D / M M / 2 Y<br>☐ Not in PICU	Day 20<br>D D / M M / 2 Y<br>☐ Not in PICU	Day 21<br>D D / M M / 2 Y<br>☐ Not in PICU	Day 22<br>D D / M M / 2 Y<br>☐ Not in PICU
No Support								
Respiratory	Invasive respiratory support							
	Any NIV (including HFNC)**							
Cardiovascular Interventions								
Corticosteroids given for cardiovascular instability								
Mechanical cardiovascular support								
Renal replacement therapy								
Plasma filtration or exchange								

		Day 23<br>D D / M M / 2 Y<br>☐ Not in PICU	Day 24<br>D D / M M / 2 Y<br>☐ Not in PICU	Day 25<br>D D / M M / 2 Y<br>☐ Not in PICU	Day 26<br>D D / M M / 2 Y<br>☐ Not in PICU	Day 27<br>D D / M M / 2 Y<br>☐ Not in PICU	Day 28<br>D D / M M / 2 Y<br>☐ Not in PICU	Day 29<br>D D / M M / 2 Y<br>☐ Not in PICU	Day 30<br>D D / M M / 2 Y<br>☐ Not in PICU
No Support									
Respiratory									
	Any NIV (including HFNC)**								
Cardiovascular Interventions									
Corticosteroids given for cardiovascular instability									
Mechanical cardiovascular support									
Renal replacement therapy									
Plasma filtration or exchange									

** NIV= Non Invasive Ventilation
HFNC = High Flow Nasal Cannula

DEFINITIONS: ORGAN SUPPORT

Invasive Respiratory Support
- Invasive mechanical ventilation i.e. must be intubated and ventilated
- Includes mechanical ventilation via an endotracheal tube or via tracheostomy
- Includes High Frequency Oscillation Ventilation (HFOV)

Any NIV (Non-invasive mechanical Ventilation)
- CPAP / BiPAP via mask (facial or nasal mask)
- High Flow Nasal Oxygen via nasal cannula (HFNC)

Do not include (in either group)
- Nasopharyngeal airway without ventilation
- Supplementary oxygen therapy alone

Cardiovascular
- Continuous inotrope/vasodilator/ prostaglandin infusion
- CPR
- Anti-arrhythmic therapy

Corticosteroids given for cardiovascular instability

Mechanical Cardiovascular Support
- ECMO
- Vascular assist device
- Aortic balloon pump

Renal Replacement Therapy
- Peritoneal dialysis
- Haemofiltration
- Haemodialysis

Plasma filtration or exchange

Signature: ____________ **Completed by:** (print name) ____________ **Date completed:** D D / M M / 2 0 2 Y

Outcomes Functional status at PICU discharge

Date of assessment: D D / M M / 2 0 2 Y

Trial ID ☐☐☐☐☐☐

Not applicable, patient died NA

Paediatric Cerebral Performance Category (PCPC)

Score (Select One)	Category	Description
1	Normal	Age-appropriate level of functioning<br>Pre-school child: developmentally appropriate<br>School-aged child: can attend regular school classroom
2	Mild disability	Conscious, alert and able to interact at age-appropriate level<br>Pre-school child: may have minor developmental delays<br>School-aged child: can attend regular classroom, but grade is not appropriate for age, or child is likely to fail appropriate grade because of cognitive difficulties
3	Moderate disability	Conscious, below age-appropriate functioning<br>Neurologic disease that is not controlled and severely limits activities<br>Pre-school child: delayed for most of their activities of daily living<br>School-aged child: Sufficient cerebral function for age-appropriate independent activities of daily life, must attend special education classroom because of cognitive difficulties and/or learning deficits
4	Severe disability	Conscious<br>Pre-school child: delayed for most of their activities of daily living and excessively dependent on others for the provision of activities of daily living<br>School-aged child: may be so impaired as to be unable to attend school, dependent on others for daily support because of impaired brain function
5	Coma or vegetative state	Any degree of coma<br>Unaware, even if awake in appearance, without interaction with environment<br>Cerebral unresponsiveness and no evidence of cortical function (eg. not aroused by verbal stimuli)<br>Possibility of some reflective response, spontaneous eye-opening and sleep-wake cycles

Paediatric Overall Performance Category (POPC)

Score (Select One)	Category	Description
1	Good overall performance	PCPC is classified as Normal<br>Healthy, alert and capable of normal age-appropriate activities of daily life<br>Medical and physical problems do not interfere with normal activity
2	Mild overall disability	PCPC classified as Mild Disability<br>Minor chronic physical or medical problems present minor limitations but are compatible with normal life (eg. asthma)<br>Pre-school child: has a physical disability consistent with future independent functioning (eg. a single amputation) and is able to perform the majority of age-appropriate activities of daily living
3	Moderate overall disability	PCPC classified as Moderate Disability<br>Possibility of moderate disability from non-cerebral systems dysfunction alone or with cerebral system dysfunction<br>Pre-school child: delayed for most of their activities of daily living<br>School-aged child: conscious and performs independent activities of daily life but is physically disabled (eg. cannot participate in competitive physical activities)
4	Severe overall disability	PCPC classified as Severe Disability<br>Possibility of severe disability from non-cerebral systems dysfunction alone or with cerebral system dysfunction<br>Pre-school child: delayed for most of their activities of daily living <u>and</u> excessively dependent on others for the provision of activities of daily living<br>School-aged child: dependent on others for most activities of daily living
5	Coma or vegetative state	PCPC classified as coma/vegetative

Signature ☐ **Completed by** (print name) ☐ **Date completed:** D D / M M / 2 0 2 Y

Outcomes - at hospital discharge

Trial ID | | | | | | |

PRESSURE

Successful Extubation

Date/time of first successful extubation[1]: D D / M M / 2 0 2 Y H H : M M (24-hour clock)

[1]Defined as extubation for at least 48 hours without reintubation

Vasoactive treatment

Total number of episodes of vasoactive drugs[2] | | |

Total number of days on vasoactive drugs: | | | |

[2]An episode is a period in which vasoactive drugs are administered continuously or with interruptions of <24hrs. If restarted after >24hrs interruption this would be defined as a new episode. There may be multiple episodes per PICU admission

Discharge from your PICU

Status: ○ Alive ○ Dead

Date/Time: D D / M M / 2 0 2 Y H H : M M (24-hour clock)

Discharge Method *(tick one)*

- ○ Patient discharged on clinical advice or with clinical consent
- ○ Patient discharged themselves
- ○ Patient was discharged by a relative or advocate
- ○ Patient discharged by mental health review tribunal, Home Secretary or Court
- ○ Unknown

Location following discharge from your PICU

Select one of the below:

○ Hospital →

Hospital*	***Location***[#]	***Date of admission***
		D D / M M / 2 0 2 Y
		D D / M M / 2 0 2 Y
		D D / M M / 2 0 2 Y
		D D / M M / 2 0 2 Y
		D D / M M / 2 0 2 Y

* **Hospital:** S = Same O = Other

Location: P = PICU O = Other

○ Home

○ Other, please specify: ____________

Ultimate discharge from Hospital

Status: ○ Alive ○ Dead

Date/Time: D D / M M / 2 0 2 Y H H : M M (24-hour clock)

Co-enrolment(s)

Please record any other interventional trials to which the patient was enrolled

Completed by: (print name) ____________

Signature: ____________ **Date completed:** D D / M M / 2 0 2 Y

Outcomes - Survival Status

Trial ID | | | | | | |

To be completed at 30 days, 90 days and 12 months from randomisation

30 days

- ○ Alive → Date status confirmed: D D / M M / 2 0 2 Y
- ○ Dead → Date of death D D / M M / 2 0 2 Y
- ○ Unable to ascertain → Date last known alive D D / M M / 2 0 2 Y

Please record reason unable to ascertain survival status:

90 days

- ○ Alive → Date status confirmed: D D / M M / 2 0 2 Y
- ○ Dead → Date of death D D / M M / 2 0 2 Y
- ○ Unable to ascertain → Date last known alive D D / M M / 2 0 2 Y

Please record reason unable to ascertain survival status:

12 months

- ○ Alive → Date status confirmed: D D / M M / 2 0 2 Y
- ○ Dead → Date of death D D / M M / 2 0 2 Y
- ○ Unable to ascertain → Date last known alive D D / M M / 2 0 2 Y

Please record reason unable to ascertain survival status:

Completed by: (print name)

Signature:

Date completed: D D / M M / 2 0 2 Y

pressure@icnarc.org

Consent/Assent

Trial ID | | | | | | |

PRESSURE

Consent

Parent/Guardian approached for consent
(tick all that apply):

In person (F) By post (P)
Deemed not appropriate for approach (N) → reason:

If in person:
Date first approached D D / M M / 2 0 2 Y

If postal approach:
Date of phone call D D / M M / 2 0 2 Y
Date of 1st postal approach D D / M M / 2 0 2 Y
Date of 2nd postal approach D D / M M / 2 0 2 Y
Response received from postal approach Yes (Y) No (N)

Consent obtained for: If bereaved:

Trial continuation	Yes (Y)	No (N)	N/A
Access to medical records for full dataset	Yes (Y)	No (N)	
Access to NHS digital data*	Yes (Y)	No (N)	N/A
Access to medical records for monitoring	Yes (Y)	No (N)	
Follow-up questionnaire	Yes (Y)	No (N)	N/A
Sharing of anonymised data	Yes (Y)	No (N)	
Future research	Yes (Y)	No (N)	

Parents/guardian details:

First name
Surname
Phone/mobile
Follow-up, email address
Follow-up, address

Contact preference Email (E) Post (P)

Date consent provided / refused D D / M M / 2 0 2 Y

Reason for refused (if provided)

Patient Details

*Please complete if obtained consent

First name
Surname

Date of birth D D / M M / Y Y Y Y
NHS number
Postcode

Assent

Approached for assent Yes (Y) No (N)
Assent given Yes (Y) No (N)
Date assent provided/declined D D / M M / 2 0 2 Y

Completed by:
(print name)

Signature:

Date completed: D D / M M / 2 0 2 Y

Safety Monitoring

(from randomisation to final critical care unit discharge during the treatment period)

Trial ID | | | | | | |

PRESSURE

Adverse events (specified)

	Severity*	Start date	Related#
Myocardial ischaemia		D D / M M / 2 0 2 Y	
Arrhythmia		D D / M M / 2 0 2 Y	
Digital or limb ischaemia		D D / M M / 2 0 2 Y	
Central line related blood stream infection (CLABSI)		D D / M M / 2 0 2 Y	
Thrombus related to central line insertion		D D / M M / 2 0 2 Y	
Skin necrosis related to administration of vasoactive via peripheral line		D D / M M / 2 0 2 Y	
Severe acute renal failure (KDIGO stage 3 criteria)		D D / M M / 2 0 2 Y	
Acute cerebral ischaemia or infarction		D D / M M / 2 0 2 Y	

If the specified adverse event did not occur, then record Severity as 0
If the specified adverse event occurred more than once this can be updated on MACRO

*** Severity: 0 = None, 1 = Mild, 2 = Moderate, 3 = Severe, 4 = Life-threatening, 5 = Fatal**

# Related (to study treatment): 0 = None, 1 = Unlikely, 2 = Possibly, 3 = Probably, 4 = Definitely

Adverse events (other/non-specified)**

Adverse event	Severity*	Start date	Related#
		D D / M M / 2 0 2 Y	
		D D / M M / 2 0 2 Y	
		D D / M M / 2 0 2 Y	
		D D / M M / 2 0 2 Y	
		D D / M M / 2 0 2 Y	

****Only record non-specified event if 'possibly' 'probably' or 'definitely' related to study treatment (specifically due to blood pressure or administration of vasoactive drugs)**

If severity of adverse event (specified or other/non-specified) is:
3 = Severe, 4 = Life threatening or 5 = Fatal

Please complete and upload the Serious Adverse Event Reporting Form on MACRO and email pressure@icnarc.org to notify us this has been done within 24 hours of becoming aware of the event

Do not send a copy via email

Completed by: (print name) ______________

Signature: ______________ **Date completed:** D D / M M / 2 0 2 Y

Withdrawal of Consent

Trial ID | | | | | | |

PRESSURE

Date of withdrawal of consent: D D / M M / 2 0 2 Y

Withdrawlng from:
(select all that apply)

Continued trial participation	Yes (Y) No (N)
Continued access to medical records for full dataset to be sent to ICNARC	Yes (Y) No (N)
Access to NHS digital data	Yes (Y) No (N)
Access to medical records for monitoring	Yes (Y) No (N)
Follow-up questionnaire	Yes (Y) No (N)
Sharing of anonymised data with other researchers	Yes (Y) No (N)
Being contacted about future research	Yes (Y) No (N)

Reason for withdrawal (if provided):
